# Costs and Cardiovascular Benefits of a Fourth-Generation Synchronous Telehealth Program on Mortality and Cardiovascular Outcomes for Patients With Atrial Fibrillation: Retrospective Cohort Study

**DOI:** 10.2196/48748

**Published:** 2024-01-08

**Authors:** Hao-Yun Chang, Hui-Wen Wu, Chi-Sheng Hung, Ying-Hsien Chen, Ching-Chang Huang, Li-Tan Yang, Shin-Tsyr Hwang, Jiun-Yu Yu, Jen-Kuang Lee, Yi-Lwun Ho

**Affiliations:** 1 Division of Cardiology, Department of Internal Medicine, National Taiwan University Hospital Taipei Taiwan; 2 Division of Cardiology, Department of Internal Medicine, National Taiwan University Hospital Hsin-Chu Branch Hsinchu Taiwan; 3 Graduate Institute of Clinical Medicine, College of Medicine, National Taiwan University Taipei Taiwan; 4 Cardiovascular Center, National Taiwan University Hospital Taipei Taiwan; 5 Telehealth Center, National Taiwan University Hospital Taipei Taiwan; 6 Department of Internal Medicine, National Taiwan University College of Medicine Taipei Taiwan; 7 Department of Nursing, National Taiwan University Hospital Taipei Taiwan; 8 Department of Business Administration, College of Management, National Taiwan University Taipei Taiwan; 9 Department of Laboratory Medicine, National Taiwan University College of Medicine Taipei Taiwan

**Keywords:** atrial fibrillation, cardiovascular death, fourth-generation synchronous program, ischemic stroke, telehealth

## Abstract

**Background:**

The prevalence of atrial fibrillation (AF) continues to increase in modern aging society. Patients with AF are at high risk for multiple adverse cardiovascular events, including heart failure, stroke, and mortality. Improved medical care is needed for patients with AF to enhance their quality of life and limit their medical resource utilization. With advances in the internet and technology, telehealth programs are now widely used in medical care. A fourth-generation telehealth program offers synchronous and continuous medical attention in response to physiological parameters measured at home. Although we have previously shown the benefits of this telehealth program for some patients with a high risk of cardiovascular disease, its benefits for patients with AF remains uncertain.

**Objective:**

This study aims to investigate the benefits of participating in a fourth-generation telehealth program for patients with AF in relation to cardiovascular outcomes.

**Methods:**

This was a retrospective cohort study. We retrospectively searched the medical records database of a tertiary medical center in Northern Taiwan between January 2007 and December 2017. We screened 5062 patients with cardiovascular disease and enrolled 537 patients with AF, of which 279 participated in the telehealth program and 258 did not. Bias was reduced using the inverse probability of treatment weighting adjustment based on the propensity score. Outcomes were collected and analyzed, including all-cause readmission, admission for heart failure, acute coronary syndrome, ischemic stroke, systemic embolism, bleeding events, all-cause mortality, and cardiovascular death within the follow-up period. Total medical expenses and medical costs in different departments were also compared. Subgroup analyses were conducted on ischemic stroke stratified by several subgroup variables.

**Results:**

The mean follow-up period was 3.0 (SD 1.7) years for the telehealth group and 3.4 (SD 1.9) years for the control group. After inverse probability of treatment weighting adjustment, the patients in the telehealth program had significantly fewer ischemic strokes (2.0 vs 4.5 events per 100 person-years; subdistribution hazard ratio [SHR] 0.45, 95% CI 0.22-0.92) and cardiovascular deaths (2.5 vs 5.9 events per 100 person-years; SHR 0.43, 95% CI 0.18-0.99) at the follow-up. The telehealth program particularly benefited patients comorbid with vascular disease (SHR 0.11, 95% CI 0.02-0.53 vs SHR 1.16, 95% CI 0.44-3.09; *P*=.01 for interaction). The total medical expenses during follow-up were similar in the telehealth and control groups.

**Conclusions:**

This study demonstrated the benefits of participating in the fourth-generation telehealth program for patients with AF by significantly reducing their ischemic stroke risk while spending the same amount on medical expenses.

## Introduction

In the past 3 decades, atrial fibrillation (AF) has become one of the most frequent cardiac arrhythmias worldwide. Its high disease burden has made it a significant public health issue. The worldwide incidence of AF in 2017 was estimated at 3046 million new cases, a 31% increase compared to 1997 [1]. The incidence of AF varies with age. AF affects <0.2% of individuals aged 49 years and younger, but 10%-17% of individuals aged 80 years or older [2]. Given the aging trend in societies worldwide, AF prevalence is expected to increase continuously [3]. Taiwan is also not exempt from this worldwide phenomenon. The prevalence of AF in Taiwan in 2011 stood at 1.07%, and it was projected to reach an estimated 4.01% by the year 2050 [4]. While multiple treatment modalities and strategies have been developed for AF, patients with AF are still associated with multiple adverse cardiovascular outcomes, including ischemic stroke, myocardial infarction, heart failure, cognitive decline, and even mortality [5]. An innovative modality to provide better outcomes for patients with AF is needed.

With advancements in technology, we have entered the internet era. Telemedicine has been well established as an essential part of contemporary medical practice. A fourth-generation telehealth program provides internet-based, synchronous, round-the-clock disease management and immediate response. Studies have shown that patients receiving telemedicine have better control over numerous cardiovascular risk factors, including diabetes mellitus, hypertension, and hyperlipidemia [6]. It even reduced all-cause mortality in heart failure patients [7,8]. Nevertheless, Wootton [9] conducted a literature review and argued that the evidence base for the value of telemedicine in managing chronic diseases was somewhat contradictory. Therefore, the benefits of telemedicine for a particular disease need to be evaluated. According to our annual report, the characteristics of the population that tends to use telehealth are male (66%), aged 65 years or older (53%), people with hypertension (55%), and people with coronary artery disease (53%). Our group previously showed the benefits of participating in a fourth-generation telehealth program for patients with peripheral artery disease [10]. However, the benefits to patients with AF remains to be determined.

This study aimed to investigate the benefits of participating in a fourth-generation telehealth program for patients with AF in relation to cardiovascular outcomes, including cardiovascular events, readmission, mortality, and financial costs.

## Methods

### Data Source and Patients

This was a retrospective cohort study. All patients aged 20 years or older diagnosed with cardiovascular disease between January 2007 and December 2017 were screened and had their charts reviewed. Cardiovascular disease included AF, coronary artery disease, myocardial infarction, heart failure, peripheral artery disease, stroke, and hypertension. Only patients with AF were selected for this study. Patients with AF who also participated in the fourth-generation synchronous telehealth program at the National Taiwan University Hospital (NTUH; Taipei, Taiwan) telehealth center were enrolled as the study group. Patients with AF who did not participate in the telehealth program receiving usual standard treatment were enrolled as the control group. The decision to participate in the telehealth program depended primarily on the patients and their caregivers. The cohort entry date was defined as the day of inviting the patients to participate in the telehealth program.

### Telehealth Care Program

The fourth-generation synchronous telehealth program at NTUH was an internet-based, multiaspects-integrated, remote management health care program for chronic disease. The Graduate Institute of Biomedical Electronics and Bioinformatics at the National Taiwan University developed the integrated platform. We previously reported the details of this program [11]. Briefly, this advanced telehealth program collects participants’ biometric data, including heart rate, blood pressure, oximetry data, and single-lead electrocardiography. These data are transmitted to our telehealth center daily and on demand. Nurses manage each participant by making telephone calls periodically and on demand, providing communication and health promotion. On-call cardiologists are available 24/7 to provide immediate responses and recommendations in any emergency. Following acute events, long-term medication and health care management are discussed with the patient’s primary care physician and provided to the patient. This telehealth program emphasizes education, medication adherence, prevention, and early condition deterioration recognition. It strengthens the bridge between acute events and long-term home care.

### Usual Standard Care

The patients in the control group did not receive the telehealth program but the usual standard treatment provided at our cardiovascular center outpatient clinics. Their primary care physician provided the usual standard care according to, but not limited to, the updated guidelines for managing chronic AF, guidelines for managing stable ischemic heart disease, American Diabetes Association guidelines for diabetes management, and the American Heart Association’s guidelines for lifestyle modification and primary prevention to reduce cardiovascular risks. There was no contact between the telehealth center, the primary care physician, and patients receiving the usual standard care.

### Covariates and Outcomes

The covariates were demographics (age, sex, and BMI), comorbidities (hypertension, diabetes, hyperlipidemia, coronary artery disease, chronic kidney disease, myocardial infarction, stroke history, heart failure, cancer, and peripheral arterial occlusive disease), “congestive heart failure, hypertension, age, diabetes mellitus, previous stroke or transient ischemic accident or thromboembolism, vascular disease, age, and sex category” (CHA_2_DS_2_-VASc) score, Charlson Comorbidity Index score, vital signs (mean arterial pressure and heart rate), laboratory data (serum creatinine and low-density lipoprotein cholesterol), left ventricular systolic function, and use of 10 medication types at enrollment. The outcomes were all-cause readmission, admission for heart failure, acute coronary syndrome, ischemic stroke, systemic embolism, bleeding events (major bleeding, gastrointestinal bleeding, and intracranial hemorrhage), all-cause mortality, and death due to cardiovascular disease during follow-up. The definition of all-cause readmission and admission for heart failure encompassed any admission to NTUH, and admission to NTUH specifically under the diagnosis of heart failure, subsequent to the cohort entry date, respectively. The date and cause of death were linked by the Taiwan Death Registry database. Each patient was followed from their cohort entry date until the day of event occurrence, the end of the database (December 31, 2017), or the day of death, whichever came first. The total number of admissions and medical expenses during follow-up were also analyzed. The medical expenses were retrieved from the NTUH electronic billing system, which encompassed comprehensive records of costs associated with outpatient services, emergency department visits, and hospitalizations. The total cost used was defined as medical expenses.

### Statistical Analysis

This study reduced selection bias using inverse probability of treatment weighting (IPTW) based on a propensity score. The propensity score was the predicted probability of being in the telehealth group given certain values of the covariates using a multivariable logistic regression model. The covariates used in the propensity score calculation are listed in Table 1, where the follow-up duration is replaced by the cohort entry date. To mitigate the impact of extreme weights, we truncated the outlier weights at the 99th percentile of the weights [12]. The balance of covariates between the telehealth and nontelehealth groups was evaluated using the absolute value of the standardized difference between groups, where a value of <0.1 was considered negligible. In addition, there were some missing data (eg, BMI, vital signs, and lipid profile), which were imputed using the single expectation-maximization algorithm. The assumption of missing completely at random was evaluated using the Little test. The IPTW was conducted on the imputed data without missing values.

**Table 1:** Baseline characteristics of patients with atrial fibrillation participating or not in the telehealth program. The number of patients has been inflated after inverse probability treatment weighting (IPTW) adjustment; therefore, n values are not provided with the percentages.

		Before IPTW			After IPTW			Available number
		Telehealth (n=279)	Nontelehealth (n=258)	STD^a^	Telehealth	Nontelehealth	STD	
Age (years), mean (SD)		68.0 (13.1)	69.2 (13.2)	–0.09	68.3 (13.0)	68.4 (13.2)	–0.01	537
Male, n (%)		156 (55.9)	157 (60.9)	–0.10	58.3	57.1	0.02	537
BMI (kg/m^2^), mean (SD)		24.4 (4.4)	24.4 (3.7)	–0.01	24.4 (4.3)	24.4 (3.6)	0.02	522
**Comorbid condition, n (%)**								537
	Hypertension	148 (53)	146 (56.6)	–0.07	55	54	0.02	
	Diabetes mellitus	78 (28)	63 (24.4)	0.08	27.7	27	0.02	
	Hyperlipidemia	95 (34.1)	84 (32.6)	0.03	32.9	31.9	0.02	
	Coronary artery disease	113 (40.5)	126 (48.8)	–0.17	44.4	44.6	<0.01	
	Chronic kidney disease	36 (12.9)	34 (13.2)	–0.01	13.3	13.3	<0.01	
	Old myocardial infarction	12 (4.3)	19 (7.4)	–0.13	7	6.4	0.03	
	Ischemic stroke	61 (21.9)	57 (22.1)	–0.01	21.4	21.1	0.01	
	Hemorrhagic stroke	2 (0.7)	3 (1.2)	–0.05	1.4	1.1	0.03	
	Transient ischemic attack	10 (3.6)	9 (3.5)	0.01	3.6	3.9	–0.01	
	Any stroke	62 (22.2)	59 (22.9)	–0.02	21.9	21.9	<0.01	
	Heart failure hospitalization	90 (32.3)	102 (39.5)	–0.15	35.5	36.2	–0.02	
	Cancer	29 (10.4)	17 (6.6)	0.14	8.8	7.7	0.04	
	Peripheral arterial occlusive disease	17 (6.1)	29 (11.2)	–0.18	8.7	8.8	<0.01	
CHA_2_DS_2_-VASc^b^ score, mean (SD)		3.4 (2.2)	3.6 (2.0)	–0.11	3.4 (2.1)	3.5 (2.0)	–0.03	
Charlson Comorbidity Index score, mean (SD)		1.78 (1.78)	1.83 (1.78)	–0.03	1.9 (1.9)	1.8 (1.8)	0.02	
**Vital sign, mean (SD)**								507
	Mean arterial pressure (mmHg)	88.3 (11.4)	89.5 (14.3)	–0.09	89.0 (10.8)	88.6 (14.4)	0.03	
	Heart rate (beat/min)	73.5 (14.0)	73.7 (16.0)	–0.01	73.5 (13.4)	73.6 (15.3)	–0.01	
**Laboratory data, mean (SD)**								
	Creatinine (mg/dL)	1.3 (1.3)	1.5 (1.5)	–0.12	1.38 (1.47)	1.37 (1.40)	0.01	536
	eGFR^c^ (mL/min/1.73 m^2^)	70.2 (27.6)	67.9 (28.8)	0.08	67.7 (27.6)	70.1 (29.0)	–0.08	536
	LDL-C^d^ (mg/dL)	97.2 (34.1)	96.2 (30.8)	0.03	96.5 (26.4)	96.2 (25.6)	0.01	360
**LVEF^e^ (%), mean (SD)**								
	Teich	61.7 (13.7)	60.9 (13.6)	0.06	61.4 (13.4)	61.3 (13.6)	0.01	407
	MOD-sp4^f^	44.0 (12.4)	43.8 (12.2)	0.02	43.4 (11.9)	44.2 (11.9)	–0.07	122
	sp4-el^g^	43.7 (12.3)	43.4 (13.1)	0.02	43.2 (12.0)	43.4 (12.9)	–0.02	103
**Medication, n (%)**								537
	ACEI^h^ or ARB^i^	140 (50.2)	140 (54.3)	–0.08	53	52.3	0.01	
	β-blocker	160 (57.3)	134 (51.9)	0.11	55	55.2	<0.01	
	dCCB^j^	102 (36.6)	105 (40.7)	–0.09	38.9	38	0.02	
	Diuretics	152 (54.5)	144 (55.8)	–0.03	54.5	54.9	–0.01	
	Aspirin	97 (34.8)	106 (41.1)	–0.13	37.1	37.8	–0.02	
	Clopidogrel or cilostazol	43 (15.4)	55 (21.3)	–0.15	18.2	19.2	–0.03	
	Oral anticoagulants	152 (54.5)	116 (45)	0.19	50.3	49.6	0.01	
	Oral hypoglycemic agents	52 (18.6)	37 (14.3)	0.12	16.8	16.3	0.01	
	Insulin	12 (4.3)	7 (2.7)	0.09	3.8	4.2	–0.02	
	Statin	76 (27.2)	73 (28.3)	–0.02	28.7	28.1	0.01	
Follow-up year, mean (SD)		3.0 (1.7)	3.4 (1.9)	–0.25	3.2 (1.8)	3.2 (1.7)	–0.01	

^a^STD: standardized difference.

^b^CHA_2_DS_2_-VASc: congestive heart failure, hypertension, age, diabetes mellitus, previous stroke or transient ischemic accident or thromboembolism, vascular disease, age, and sex category.

^c^eGFR: estimated glomerular filtration rate.

^d^LDL-C: low-density lipoprotein cholesterol.

^e^LVEF: left ventricular ejection fraction.

^f^MOD-sp4: method of disc-single plane 4 chamber view.

^g^sp4-el: single plane 4 chamber view-ellipsoid model.

^h^ACEi: angiotensin-converting enzyme inhibitor.

^i^ARB: angiotensin receptor blocker.

^j^dCCB: dihydropyridine calcium channel blocker.

The incidence of outcomes during follow-up was calculated using the incidence density, expressed as the number of events per 100 person-years. The absolute risk difference between the groups was also calculated in the IPTW-adjusted cohort. Furthermore, the risks of outcomes during follow-up were compared between groups using the Cox proportional hazards model for fatal outcomes (eg, mortality) or the Fine-Gray subdistribution hazard model for nonfatal outcomes (eg, ischemic stroke). The outcomes were assessed during the follow-up period. The average number of all-cause readmissions during follow-up between groups was compared using a Poisson model, which treated the logarithm of follow-up duration as an offset variable. The accumulated medical expenditures during follow-up were compared between groups using a linear regression model. The study group was the only explanatory factor in these regression models.

A subgroup analysis was conducted on ischemic stroke outcome stratified by several subgroup variables, including CHA_2_DS_2_-VASc score (<4 vs ≥4), sex, age (<75 years vs ≥75 years), hypertension, heart failure, diabetes, previous ischemic stroke, vascular disease, and use of oral anticoagulants. A 2-sided *P* value of <.05 was considered statistically significant. However, the clinical significance of subgroup analyses was loosened to a *P* value of <.10 because the interaction test was known to be conservative [13,14]. All statistical analyses were performed using SAS (version 9.4; SAS Institute).

### Ethical Considerations

This study was approved by the institutional review board of NTUH (201804072RINA). This study was conducted under the relevant regulation and protocols.

## Results

### Patients’ Demographics and Clinical Characteristics

This study screened 5062 patients, of whom 537 had AF and were selected for analysis. Among them, a total of 279 and 258 patients were in the telehealth and nontelehealth groups, respectively. Their detailed demographics and clinical characteristics are listed in Table 1. The mean age was 68.0 (SD 13.1) years in the telehealth group and 69.2 (SD 13.2) years in the nontelehealth group. Before IPTW, patients in the telehealth group were less likely to be male; had lower prevalence of coronary artery disease, myocardial infarction, heart failure hospitalization, and peripheral arterial occlusive disease; had lower CHA_2_DS_2_-VASc scores; had lower serum creatinine levels; were more likely to take β-blockers, oral anticoagulants, and oral hypoglycemic agents; and were less likely to take aspirin and clopidogrel or cilostazol. Patients in the telehealth group had a higher prevalence of cancer. However, after IPTW adjustment, the characteristics were well-balanced between the groups, with all the absolute standardized difference values being <0.1. The mean follow-up time was 3.0 (SD 1.7) years for the telehealth group and 3.4 (SD 1.9) years for the nontelehealth group. In addition, the assumption of missing completely at random was held given the insignificant Little test.

### Prognosis of Patients With AF Participating in the Telehealth Program

**Figure 1:**
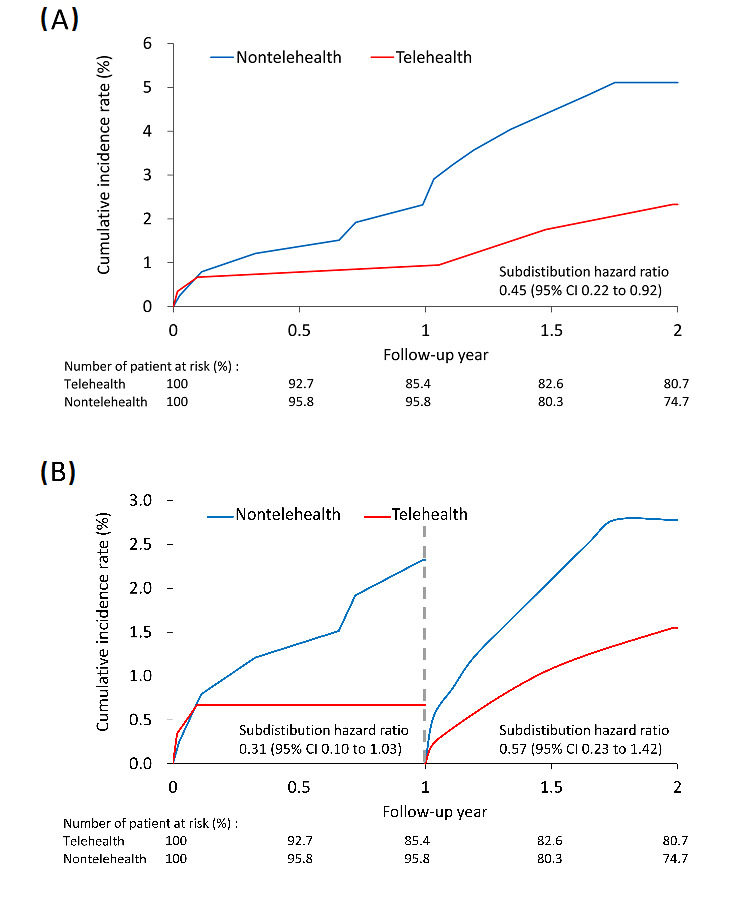
The cumulative incidence of ischemic stroke in patients with atrial fibrillation participating (red line) and not participating (blue line) in the telehealth program in the inverse probability treatment weighting–adjusted cohort over (A) the follow-up period and (B) in each follow-up year.

**Figure 2:**
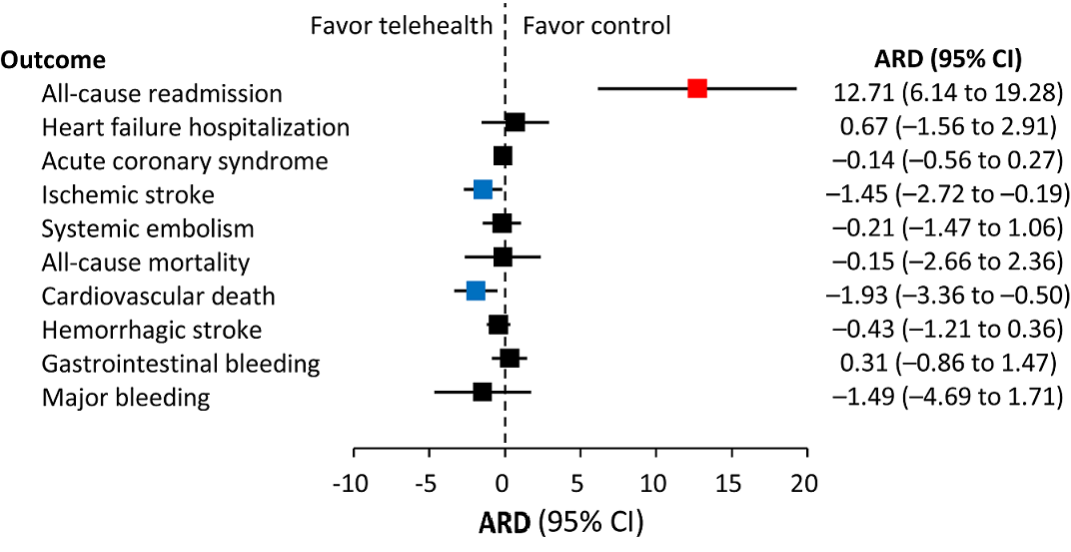
The absolute risk difference of each outcome during 2-year follow-up in patients with atrial fibrillation participating and not participating in the telehealth program in the inverse probability treatment weighting–adjusted cohort. ARD: absolute risk difference.

After IPTW adjustment, the all-cause admission rate during the follow-up period was significantly higher in the telehealth group (50.4 vs 39.7 events per 100 person-years; subdistribution hazard ratio [SHR] 1.38, 95% CI 1.15-1.65). Notably, the incidence of ischemic stroke was significantly lower in the telehealth group (2.0 vs 4.5 events per 100 person-years; SHR 0.45, 95% CI 0.22-0.92). In addition, cardiovascular death was also significantly lower in the telehealth group (2.5 vs 5.9 events per 100 person-years; SHR 0.43, 95% CI 0.18-0.99; Table 2). The cumulative incidence of ischemic stroke was consistently lower in the telehealth group, deviating further from the nontelehealth group over time (Figure 1A). Separately considering each follow-up year, the cumulative incidence of ischemic stroke was consistently lower in the telehealth group in both the first and second years of follow-up (Figure 1B). The absolute risk difference for each outcome is shown in Figure 2.

**Table 2:** Clinical outcomes of patients with atrial fibrillation at the follow-up. The number of patients has been inflated after inverse probability treatment weighting adjustment; therefore, n values are not provided with the percentages.

Outcome	Telehealth		Nontelehealth		ARD^a^ (95% CI)	Ratio of telehealth, HR^b^ or SHR^c^ (95% CI)	*P* value
	%	ID^d^ (95% CI)^e^	%	ID (95% CI)^e^			
All-cause readmission	50.4	43.1 (38.0 to 48.2)	39.7	30.4 (26.3 to 34.5)	12.71 (6.14 to 19.28)	1.38^f^ (1.15 to 1.65)	.001
Heart failure hospitalization	9.6	6.0 (4.4 to 7.6)	8.8	5.3 (3.8 to 6.9)	0.67 (–1.56 to 2.91)	1.12^f^ (0.76 to 1.67)	.57
Acute coronary syndrome	0.2	0.13 (–0.10 to 0.37)	0.5	0.28 (–0.06 to 0.62)	–0.14 (–0.56 to 0.27)	0.48^f^ (0.06 to 4.13)	.50
Ischemic stroke	2	1.2 (0.5 to 1.9)	4.5	2.6 (1.6 to 3.7)	–1.45 (–2.72 to –0.19)	0.45^f^ (0.22 to 0.92)	.03
Systemic embolism	3	1.8 (0.9 to 2.6)	3.4	2.0 (1.1 to 2.9)	–0.21 (–1.47 to 1.06)	0.90^f^ (0.46 to 1.77)	.77
Major bleeding	5.4	5.8 (3.7 to 7.9)	6.7	7.3 (4.9 to 9.7)	–1.49 (–4.69 to 1.71)	0.72^f^ (0.45 to 1.17)	.19
Gastrointestinal bleeding	2.9	1.8 (0.9 to 2.6)	2.5	1.5 (0.7 to 2.2)	0.31 (–0.86 to 1.47)	1.23^f^ (0.59 to 2.53)	.58
Intracranial hemorrhage	0.9	0.53 (0.06 to 1.00)	1.7	0.95 (0.32 to 1.58)	–0.43 (–1.21 to 0.36)	0.56^f^ (0.18 to 1.69)	.30
All-cause mortality	12.7	7.5 (5.7 to 9.2)	13.3	7.6 (5.9 to 9.4)	–0.15 (–2.66 to 2.36)	0.97^g^ (0.58 to 1.65)	.92
Cardiovascular death	2.5	1.5 (0.7 to 2.3)	5.9	3.4 (2.2 to 4.6)	–1.93 (–3.36 to –0.50)	0.43^g^ (0.18 to 0.99)	.049

^a^ARD: absolute risk difference.

^b^HR: hazard ratio.

^c^SHR: subdistribution hazard ratio.

^d^ID: incidence density.

^e^100 person-years.

^f^Subdistribution hazard ratio.

^g^Hazard ratio.

### Subgroup Analysis of Ischemic Stroke

**Figure 3:**
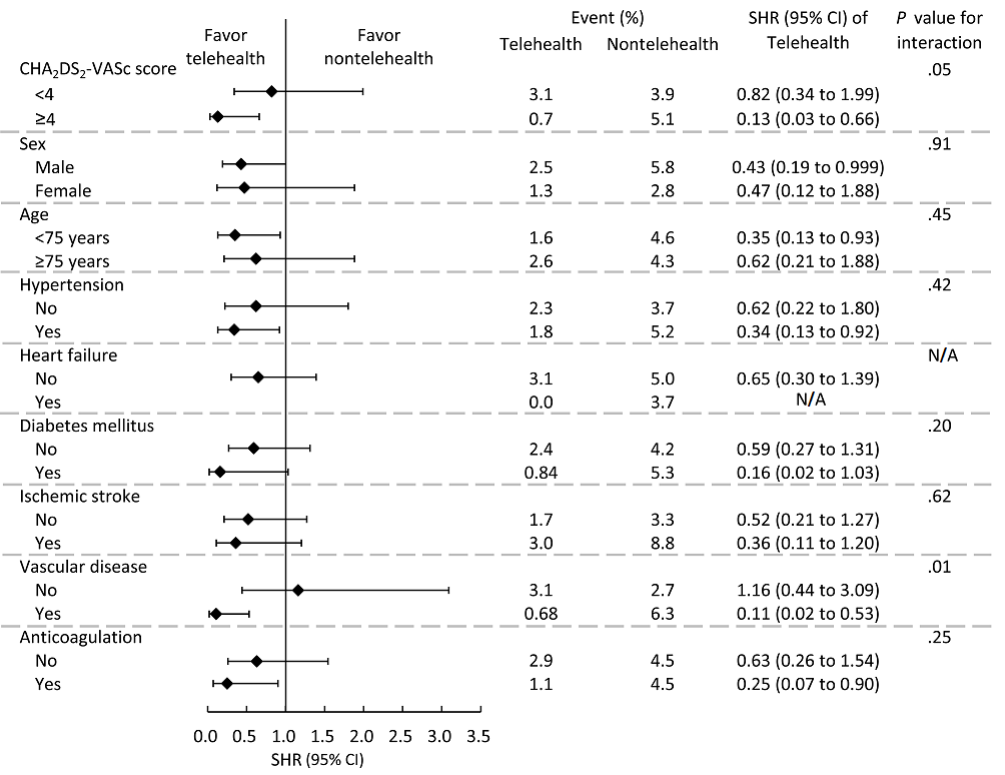
The different subgroup analysis of ischemic stroke risk during the 2-year follow-up period. The subdistribution hazard ratio (SHR) and *P* value for variable interaction are shown in the right columns. CHA2DS2-VASc: congestive heart failure, hypertension, age, diabetes mellitus, previous stroke or transient ischemic accident or thromboembolism, vascular disease, age, and sex category; NA: not applicable.

The subgroup analysis showed that the observed favorable effect in the telehealth group was more apparent in patients with CHA_2_DS_2_-VASc scores ≥4 than <4 (SHR 0.13, 95% CI 0.03-0.66 vs SHR 0.82, 95% CI 0.34-1.99; *P*=.05 for interaction) but not statistically significant. However, the observed favorable effect in the telehealth group was more pronounced in patients with than without vascular disease (SHR 0.11, 95% CI 0.02-0.53 vs SHR 1.16, 95% CI 0.44-3.09; *P*=.01 for interaction). The remaining subgroup variables had no significant interaction effects (Figure 3).

### Medical Use and Costs for Patients Participating in the Telehealth Program

Readmissions were significantly higher in the telehealth group (1.6, SD 2.3 times) than in the nontelehealth group (1.2, SD 1.7 times; rate ratio 1.35, 95% CI 1.22-1.50). Nevertheless, medical expenditure did not differ significantly between groups during the entire follow-up period, including outpatient clinic, hospitalization, emergency department, and total expenses (Table 3).

**Table 3:** Medical use and costs for patients with atrial fibrillation participating or not in the telehealth program.

Outcome		Telehealth (n=537.2), mean (SD)	Nontelehealth (n=532.9), mean (SD)	Ratio of telehealth, RR^a^ or B^b^ (95% CI)	*P* value
Number of readmissions		1.6 (2.3)	1.2 (1.7)	1.35^c^ (1.22 to 1.50)	<.001
**Medical expenditure (US$ × 10^3^)**					
	Outpatient	11.0 (10.5)	9.5 (10.1)	1.50^d^ (–0.25 to 3.24)	.09
	Hospitalization	19.4 (18.8)	19.8 (19.5)	–0.37^d^ (–3.60 to 2.87)	.83
	Emergency department	1.2 (1.5)	1.2 (1.6)	–0.02^d^ (–0.29 to 0.24)	.87
	Total	37.9 (58.6)	33.3 (35).8	4.6^d^ (–3.6 to 12.8)	.27

^a^RR: rate ratio.

^b^B: regression coefficient.

^c^Rate ratio.

^d^Regression coefficient.

## Discussion

### Principal Results

This study marked a pioneering investigation into the efficacy of implementing the fourth-generation synchronous telehealth program for patients with AF in terms of clinical outcomes. The results demonstrated a favorable effect of the telehealth program, effectively reducing the risk of stroke in patients diagnosed with AF. It also shows that the patients in the telehealth program had significantly fewer cardiovascular deaths at the follow-up. Besides, the total medical expenses during follow-up were similar in the telehealth and control groups.

### Overviews

Among all possible complications of AF, ischemic stroke is the most feared and highly related. The average annual risk of ischemic stroke in patients with AF was 4.4% [15], and AF increased the relative risk of stroke 4-5-fold [16]. Since it was frequently encountered and had severe consequences leading to disability and mortality, any method to reduce this risk has great clinical benefit and impact. This study demonstrated that participating in the telehealth program significantly decreased the stroke rate. The difference was evident as early as the first year and continuously had a favorable effect on ischemic stroke events in the second year of follow-up. By the end of the 2-year follow-up, the risk was reduced by half in the patients receiving standard care plus a telehealth program compared to those receiving standard in-person clinic follow-up only (SHR 0.45, 95% CI 0.22-0.92). The results showed that the difference between the 2 groups increased over time. The greater the adherence to the telehealth program, the greater the benefit to the patient. Similarly, Guo et al [17,18] showed that using mobile health technology for AF patient management could improve quality of life, increase medication adherence, improve patient education, and reduce the risk of rehospitalization and adverse events.

Besides ischemic stroke, the absolute risk difference for cardiovascular death also showed a favorable outcome in the telehealth group. There were significantly fewer cardiovascular deaths in the telehealth group. While the telehealth program appeared to have a negative effect in this study, causing more readmissions, it translated into better clinical outcomes by reducing ischemic stroke and cardiovascular death. There were many possible reasons for these results. The fourth-generation synchronous telehealth program provided real-time feedback on physiological measurements with periodic web-based check-ins by the nurse managers and timely interpretation by the cardiologists. Early recognition of disease deterioration, symptom development, or condition change triggered a hospital visit and possible subsequent hospitalization but ultimately contributed to better clinical outcomes. In addition, this program also facilitated timely management and increased drug adherence.

Despite many studies showing favorable telemedicine outcomes, others failed to show better clinical outcomes in patients participating in telehealth programs [9,19]. Differences in patient population enrollment, telehealth programs, and telemonitor devices across studies could explain these contradictory results. Therefore, the effectiveness of each telehealth program for a specific disease must be evaluated individually. Our group has previously reported the favorable outcomes of participating in the fourth-generation synchronous telehealth program for patients with chronic cardiovascular and peripheral artery disease [10,11]. This study demonstrated and confirmed that applying this telehealth program to patients with AF was also beneficial.

Subgroup analysis provided further insight into which population benefits from this telehealth program the most in relation to reduced ischemic stroke risk. Pre-existing vascular disease showed the strongest interaction regarding fewer stroke events. Peripheral artery disease, coronary artery disease, and cerebral vascular disease shared common risk factors with ischemic stroke and were all associated with a higher stroke rate [20-22]. It is well known that patients with higher CHA_2_DS_2_-VASc scores have an increased risk for ischemic stroke. Notably, there is a trend for clinical significance that a protective effect was observed within the telehealth group with a CHA_2_DS_2_-VASc ≥4 points, but the *P* value of .05 for interaction did not attain statistical significance. The limited sample size may also conceal the true significance. We previously demonstrated that patients with higher CHA_2_DS_2_-VASc scores had higher risks of cardiovascular admissions, but the fourth-generation telehealth program diminished the outcome difference [23]. In clinical practice, it has also been observed that patients at high risk of stroke derive the greatest benefit from stroke prevention measures [24]. Our results concurred with those findings and indicated that those with high ischemic stroke risk (comorbid with vascular disease or a high CHA_2_DS_2_-VASc score) were the most valuable target population for this telehealth program.

One of the most frequently mentioned barriers to applying telemedicine to patient care was the increasing financial cost and lack of reimbursement [25]. Some studies performed a cost-effectiveness analysis to support the extra expenditure and advocate telemedicine [26,27]. This study even showed that the fourth-generation telehealth program used for patients with AF was cost-efficient and did not increase total medical expenses. Therefore, the benefits of this telehealth program did not come at the expense of financial harm to the patients.

### Limitations and Strengths

This study had several strengths and limitations. Most published studies discussing telemedicine used a follow-up duration of only several months. The mean follow-up duration in this study was >3 years in both the control and telehealth groups. For most chronic disease complications, prevalence positively correlated with time. Some have argued that the beneficial evidence was not robust due to the short follow-up period. This issue was somewhat overcome in this study, which provided evidence regarding relative long-term clinical outcomes. However, this study was retrospective and nonrandomized, resulting in heterogeneity in the patient population and disease severity. The 2 groups were well-matched after IPTW adjustment, minimizing the possible confounding effect of clinical factors. Second, the clinical outcomes were retrieved from our hospital’s medical records and electronic billing system. If the patient received extra medical care outside of our hospital, it was not recorded. Potential resources used but not charged for were also neglected and overlooked when calculating expenditures.

### Conclusions

In summary, this study showed the benefits to patients with AF of participating in the fourth-generation synchronous telehealth program. Their stroke risk was significantly decreased, especially for those with pre-existing vascular disease and high CHA_2_DS_2_-VASc scores. There was no increase in total medical expenditure when participating in this telehealth program. Future prospective, randomized trials are needed to further confirm our findings and fully integrate them into the optimal multimodality management of patients with AF.

## Abbreviations

AF: atrial fibrillation
CHA2DS2-VASc: congestive heart failure, hypertension, age, diabetes mellitus, previous stroke or transient ischemic accident or thromboembolism, vascular disease, age, and sex category
IPTW: inverse probability treatment weighting
NTUH: National Taiwan University Hospital
SHR: subdistribution hazard ratio
